# Analysis of risk factors associated with hepatocellular carcinoma in black South Africans: 2000–2012

**DOI:** 10.1371/journal.pone.0196057

**Published:** 2018-05-02

**Authors:** Daniel Mak, Chantal Babb de Villiers, Charles Chasela, Margaret I. Urban, Anna Kramvis

**Affiliations:** 1 Hepatitis Virus Diversity Research Unit (HVDRU), Department of Internal Medicine, School of Clinical Medicine, Faculty of Health Sciences, University of the Witwatersrand, Johannesburg, South Africa; 2 Cancer Epidemiology Research Group, National Cancer Registry, National Health Laboratory Service, Johannesburg, South Africa; 3 Division of Human Genetics, Faculty of Health Sciences, University of the Witwatersrand, Johannesburg, South Africa; 4 Epidemiology and Strategic Information (ESI), HIV/AIDS, STIS and TB (HAST), Human Sciences Research Council (HSRC), Pretoria, South Africa; 5 Department of Epidemiology and Biostatistics, School of Public Health, Faculty of Health Sciences, University of the Witwatersrand, Johannesburg, South Africa; 6 Right to Care, EQUIP HEALTH, Centurion, South Africa; University of Cincinnati College of Medicine, UNITED STATES

## Abstract

**Objective:**

The aims of this study were to determine the prevalence of risk factors associated with hepatocellular carcinoma (HCC) in black adult South Africans and to estimate the size of the associated risks.

**Methods:**

A case-control analysis of 150 black South African patients (aged 18–75 years) with HCC—who were a subset of patients recruited for the Johannesburg Cancer Case Control Study 2000 to 2012—was undertaken. The association between this tumour and hepatitis B/C virus infections, and human immunodeficiency virus (HIV) mono- and co-infections was investigated. Odds ratios (ORs) and 95% confidence intervals (CI) adjusted for age, year of diagnosis, marital status, place of birth and selected modifiable risk factors were calculated.

**Results:**

HCC was significantly associated with a rural birthplace (p<0.05), being male and living in an urban area for 14 years or less. The Odds Ratio (OR) for HCC increased significantly with HBV DNA+/HBsAg+ (OR 34.5; CI:16.26–73.13), HBV DNA+/HBsAg- (OR 3.76; CI:1.79–7.92), HBV DNA level >2000 IU/ml (OR 8.55; CI:3.00–24.54) to ≥200,000 (OR 16.93; CI:8.65–33.13), anti-HCV (OR 8.98; CI:3.59–22.46), HBV DNA+/HIV+ co-infection (OR 5.36; CI:2.59–11.11), but not with HBV DNA-/HIV+ (OR 0.34; CI:0.14–0.85). We did not find a synergistic interaction between HBV and HIV. Modifiable risk factors (alcohol consumption, tobacco smoking, number of sexual partners, diabetes and hormonal contraceptive use) were nonsignificant.

**Discussion:**

A considerable portion of the HCC burden in Johannesburg and surrounding provinces falls on rural migrants to urban areas, most of whom are men. The HBV will continue to contribute to HCC incidence in older age-groups and in others who missed vaccination. Although we did not find an increased risk for HCC in HIV positive individuals this may change as life expectancy increases due to greater access to antiretroviral therapies, necessitating the addition of hepatitis virus screening to preventive medical care.

## Introduction

Hepatocellular carcinoma (HCC) is the fifth most common malignancy and second leading cause of cancer-related mortality worldwide (745,000 deaths or 9.1% of the total cancer deaths) [1]. Approximately 80% of HCC cases occur in developing regions including sub-Saharan Africa and southeast Asia [1], and is usually associated with poor prognosis (5-year survival of 14% from diagnosis) [2]. Surgical interventions reportedly have a high recurrence (81%) of HCC and a low 5-year survival rate (38%) in South Africa (SA) [3].

In sub-Saharan Africa, epidemiological studies show that the most common aetiological risk factors for HCC include hepatitis B virus (HBV), hepatitis C virus (HCV), and dietary exposure to the fungal hepatocarcinogen aflatoxin B_1_ [4]. Total aflatoxin levels in SA commercial foods are regulated by Section 15(1) of the Foodstuffs, Cosmetics and Disinfectants Act, 1972. Regardless of this, significant aflatoxin contamination does occur in stored crops such as maize and groundnuts grown and stored for family or local consumption; the problem is weather-dependent and expected to increase with increasing climate variability [5].

The World Health Organization (WHO) estimated that, in 2015, 257 million people were infected with HBV and 70 million people with HCV, of whom 60 million and 11 million resided in Africa, respectively [6]. In SA HBV is a well-recognized risk factor for HCC. HBV infection is characterized by childhood acquisition via horizontal transmission, male predominance and a high prevalence in rural-born black Africans [7]. Hepatitis B vaccine, introduced into the SA Expanded Programme on Immunisation in 1995, has been shown to effectively control the spread of HBV in children [8].

In recent decades human immunodeficiency virus (HIV) infected persons co-infected with hepatitis virus (HBV/HCV) have become a serious public health concern internationally due to a significantly higher risk of HCC development [9] and mortality [10]. Most of these studies were carried out in regions of low HBV endemicity where infections with HIV and hepatitis B/C viruses occur at more or less the same age (sexual maturity) and are mostly limited to certain high-risk groups [11]. It is unknown whether the effects of HIV on HCC risk are the same when it is acquired years after a chronic HBV infection is established, as is the case in sub-Saharan African countries [12].

Thus, the aim of this study was to determine the prevalence of risk factors for HCC development in black South African adults born prior to the introduction of routine HBV vaccination in 1995, as part of the Expanded Programme on Immunisation [8]. These factors included both viral factors (HBV/HCV; HIV) and potentially modifiable risk factors including alcohol consumption, tobacco smoking, self-reported diabetes and hormonal contraception use.

## Materials and methods

### Study design and setting

This retrospective analysis consisted of a subset of participants enrolled in the Johannesburg Cancer Case Control Study (JCCCS), a study that recruited self-identified black African (not mixed ancestry) cancer patients in the greater Johannesburg, Gauteng province, public referral hospitals that offer oncology services. Prior to commencement of cancer treatment, a written or witnessed verbal consent was obtained for interview and for having blood drawn for future assessment of factors of interest. Face to face interviews were conducted by trained research nurses who assigned a study number to participants. Each participant also gave consent to have records reviewed by the research nurses who collected the required information for the cancer diagnosis confirmation. Blood specimens were independently anonymously tested for HIV infection and stored at the National Health Laboratory Service as previously described [13]. All patient information provided to researchers was only identified by the study number.

The study protocol conforms to the ethical guidelines of the 1975 Declaration of Helsinki as reflected in *a priori* approval granted by the University of the Witwatersrand Human Research Ethics Committee (Medical) (M150239; M140271).

### Eligibility criteria

Newly diagnosed with primary HCC (ICD-O: 8170/3 and ICD10: C22), with no prior history of any cancer, was enrolled from 1 September 2000 to 31 December 2012. A total of 158 HCC cases, all with available stored pretreatment serum, were included. Fifty-one (34%) were confirmed by histology, 31 (20.7%) by cytology, 57 (38%) by elevated alpha fetoprotein levels (>450 ng/ml) and/or ultrasound (4) or CT scan (1), with the remaining cases (7.3%) confirmed as HCC by physicians. In the time period of the analysis only 8 potential HCC cases were excluded: 4 because patient files for confirmation of diagnosis could not be located, 3 because only a discharge or transfer letter indicating the HCC diagnosis was available, and 1 because the tumour was a secondary cancer.

For each HCC case three matched controls were randomly selected from the JCCCS database, matched by recruitment hospital, sex, and age at the time of interview (± 3 years). Following previously published criteria controls were included on the basis of confirmed malignant neoplasms that are not known to have a possible infectious cause [14]. Cancers with ill-defined, secondary and unspecified sites of carcinoma were also excluded. Twelve (2.7%) potential controls could not be used because sera were depleted.

It is highly unlikely that any of the participants were vaccinated against HBV because all participants were born prior to 1995, the year HBV vaccination, at 6, 10 and 14 weeks of age was introduced into the South African Expanded Programmed on Immunisation [8].

### Laboratory testing

Enzyme-linked immunosorbent assay kits were used to test for serological markers of anti-HBc (Murex^®^ Anti-HBc Total, Abbott Diagnostics, UK) and anti-HCV (Murex anti-HCV 4.0 ELISA, Abbott Diagnostics, UK). All HBV-DNA positive samples were tested for HBsAg (Murex HBsAg v3.0, Abbott Diagnostics, UK), and all HBsAg-positive further tested for HBeAg (ETI-EBK PLUS; DiaSorin).

HBV-DNA was extracted from 200 μl of serum using a QIAamp^®^ DNA Blood Mini Kit (Qiagen, Germany), according to the manufacturer’s instructions. HBV-DNA detection and quantification were performed as described previously [15]. Briefly, quantitative real-time PCR (RT-PCR) was performed in a 25 μl total volume using 12.5μl of 2X EagleTaq Universal Master Mix with ROX (Roche Applied Science, Basel, Switzerland), 10 μM HBV-Taq1 forward primer, 10μM HBV-Taq2, 10 μM BS-1 probe and 2 μl of DNA. Thermal cycling was performed in a CFX96 Touch^™^ Real-Time PCR Detection System (Bio-Rad, Inc., Hercules, CA) as follows: 1 cycle of 2 min at 50°C and 10 min at 95°C, followed by 45 cycles of 95°C for 15 sec and 60°C for 1 min. HBV-DNA concentration was calculated using a standard of cloned plasmid full-genome HBV-DNA, with concentrations ranging from 5x10^2^ to 5x10^7^ IU/ml. Calibration of this standard was confirmed by comparison with an World Health Organization (WHO) International HBV-DNA Standard (product code 97/750 NIBSC; Hertfordshire, UK). The averaged copies/ml value was divided by 4.7 to convert copies/ml to IU/ml, with a detection limit of ~20 IU/ml or 94 copies/ml [16]. The blank, positive controls, negative controls, and samples were all tested in duplicate; the standard curve samples were tested in triplicate.

### Risk factor classification

Modifiable risk factors—alcohol consumption, tobacco smoking and hormonal contraceptive use—included in this study have been previously described [17, 18]. Briefly, current smokers, including those who had stopped smoking within 5 years of date of interview, were classified according to the daily number of cigarettes smoked, and were subdivided into light (1–14 g/day) and heavy (≥15 g/day) smokers. Those who stopped smoking more than 5 years prior to the date of interview were classified as former smokers. Current alcohol drinkers were subdivided into categories based on the frequency of alcohol consumed per week assuming 1 serving of an alcoholic beverage contains 10g of ethanol: non-drinkers (<1 drink/week); moderate drinkers (1–7 drinks/week for women, 1–14 drinks/week for men; heavy drinkers– 8 or more drinks/week or 4 or more drinks in a single occasion for women, 15 or more drinks/week or 5 or more drinks in a single occasion for men [19]. HBV-DNA levels were divided into HBV-DNA categories according to the South African guidelines for the management of chronic HBV infection [20]. Occult HBV infection (OBI) was characterised by the presence of HBV-DNA in HBsAg-negative individuals with or without serological markers of HBV infection [21]. Use of antiretroviral therapy (ART) was only included in the questionnaire from November 2004; thus this information was excluded from analysis.

### Statistical analyses

Continuous variables such as the age of participants were summarized as means (standard deviation); HBV-DNA levels (>20 IU/mL) were summarized as medians (interquartile range). Chi-square and Fisher’s exact (for sparse data) were used to evaluate differences among and between the case and the control groups. The Shapiro-Wilk test was used to test for normal distribution of quantitative values. All non-normal distributions were analysed with the Mann-Whitney test. Conditional logistic regression models were used to evaluate the impact of independent predictors of the outcome as odds ratios (OR) with 95% confidence intervals (CI) and assessment of potential confounding factors including sex, age group, country and province of birth, number of years lived in urban area (0–14, 15–29 and ≥30 years), place of birth (urban or rural), alcohol consumption, HIV status and anti-HCV positivity. Adjustment of other potential HCC confounders, including marital status, smoking, self-reported diabetes, hormonal contraception use and the number of lifetime sexual partners, did not significantly change the estimates significantly and were therefore not included in the model. Statistical significance was defined as p-value<0.05. All analysis was done using STATA software, version 13.1 (StataCorp. 2013. *Stata Statistical Software*: *Release 13*. College Station, TX: StataCorp LP).

## Results

Table 1 shows the demographic characteristics and viral infection status of the HCC and control participants. The majority of participants were born in SA (88.9%) and close to two thirds were born outside the Gauteng Province (60.4%). HCC cases were more likely to be male (74%) and rural-born (65.3%, p = 0.03). The male-to-female ratio was higher for the rural-born (4.2:1) than urban-born participants (1.6:1), and in the older population (>40 years old) born in rural areas (5:1), compared to their younger counterparts (≤40 years old) (3:1). In contrast, urban-born HCC cases reported a more equal male-to-female ratio in the two age groups (1.5:1 and 1.7:1 for >40 and ≤40 years old, respectively). The difference between the HCC cases and controls in terms of the number of years recently lived in an urban environment was also statistically significant, with approximately half of all HCC cases (48%) having lived in an urban area for only 0–14 years, compared to about a third of controls (34.9%, p = 0.01). The prevalence of HBV DNA and anti-HCV antibodies was significantly higher in HCC cases than in the controls (p<0.001), while HIV-positivity did not differ statistically between the groups (18.7% vs 25.8%, p = 0.08).

**Table 1:** Demographic and virological characteristics: HCC cases and age- and sex-matched control participants from the Johannesburg Cancer Case Control Study, 2000 to 2012.

Characteristic	Total	HCC	Control	p-value
	N (%)	N (%)	N (%)	
**Age**				0.97
18–29	65 (11.1)	15 (10.0)	50 (11.4)	
30–39	150 (25.5)	37 (24.7)	113 (25.8)	
40–49	142 (24.1)	37 (24.7)	105 (24.0)	
50–59	120 (20.4)	33 (22.0)	87 (19.9)	
≥ 60	111 (18.9)	28 (18.7)	83 (18.9)	
*Missing Data*	0 (0.0)	0 (0.0)	0 (0.0)	
mean (SD)	45.8±13.3	46.1±13.3	45.7±13.3	
**Sex**				1.00
Female	153 (26.0)	39 (26.0)	114 (26.0)	
Male	435 (74.0)	111 (74.0)	324 (74.0)	
*Missing Data*	0 (0.0)	0 (0.0)	0 (0.0)	
**Year of Interview**				0.51
2000–2004	192 (32.7)	48 (32.0)	144 (32.9)	
2005–2008	194 (33.0)	45 (30.0)	149 (34.0)	
2009–2012	202 (34.4)	57 (38.0)	145 (33.1)	
*Missing Data*	0 (0.0)	0 (0.0)	0 (0.0)	
**Marital Status**				0.37
Married/living as married	362 (61.6)	99 (60.0)	263 (60.0)	
Separated	60 (10.2)	10 (6.7)	50 (11.4)	
Single/ Never Married	111 (18.9)	28 (18.7)	83 (18.9)	
Widowed	53 (9.0)	13 (8.7)	40 (9.1)	
*Missing Data*	2 (0.3)	0 (0.0)	2 (0.5)	
**Country of Birth**				<0.001
Outside South Africa	65 (11.1)	29 (19.3)	36 (8.2)	
South Africa	523 (88.9)	121 (80.7)	402 (91.8)	
*Missing Data*	0 (0.0)	0 (0.0)	0 (0.0)	
**Province of Birth**				0.21
Gauteng	207 (39.8)	42 (28.0)	165 (37.7)	
Other Provinces	316 (60.4)	79 (52.7)	237 (54.1)	
*Missing Data*	0 (0.0)	0 (0.0)	0 (0.0)	
**Place of Birth**				0.03
Rural	346 (57.7)	98 (65.3)	241 (55.2)	
Urban	253 (42.2)	52 (34.7)	196 (44.9)	
*Missing Data*	1 (0.2)	0 (0.0)	1 (0.2)	
**Place of Residence**				0.15
Rural	55 (9.4)	11 (7.3)	44 (10.0)	
Urban	532 (90.5)	138 (92.0)	394 (90.0)	
*Missing Data*	1 (0.2)	1 (0.7)	0 (0.0)	
**Years lived in Urban**				0.01
0–14 years	225 (38.3)	72 (48.0)	153 (34.9)	
15–29 years	113 (19.2)	27 (18.0)	86 (19.6)	
≥30 years	247 (42.0)	50 (33.3)	197 (45.0)	
*Missing Data*	3 (0.5)	1 (0.7)	2 (0.5)	
**HIV Status**				0.08
Positive	141 (24)	28 (18.7)	113 (25.8)	
Negative	447 (76)	122 (81.3)	325 (74.2)	
*Missing Data*	0 (0.0)	0 (0.0)	0 (0.0)	
**HBV DNA Status**				<0.001
Positive	136 (23.1)	80 (53.3)	56 (12.8)	
Negative	452 (76.9)	70 (46.7)	382 (87.2)	
*Missing Data*	0 (0.0)	0 (0.0)	0 (0.0)	
**Anti-HCV Status**				<0.001
Positive	32 (5.4)	19 (12.7)	13 (3.0)	
Negative	550 (93.5)	131 (87.3)	419 (95.7)	
*Missing Data*	6 (1.0)	0 (0.0)	6 (1.4)	

Of all the 588 participants, 315 (53.6%) were anti-HBc-positive and 136 (23.1%) were HBV DNA-positive (14.1% and 8.8% of HBV DNA-positive were HBsAg-positive HBsAg-negative, respectively). Of the 150 HCC cases, HBsAg positivity was significantly higher in men than in women (77.5% vs 22.5%, respectively). HBsAg- and HBeAg-positivity was significantly different between HCC cases and controls (HBsAg: 41.3% and 4.8%, respectively, p<0.001; HBeAg: 12% and 0.9%, respectively, p<0.05). Table 2 suggests that the increase in the risk for HCC was associated with some combination of HBV markers—anti-HBc, HBV-DNA and/or HBsAg. The multivariate analysis of HBV markers did not meaningfully differ from those observed in univariate analysis. Relative to those who tested negative for all markers, the risk for HCC among HBV DNA-positive participants who were HBsAg-positive or HBsAg-negative, was estimated at OR 34.48 (95% CI: 16.26–73.13) and OR 3.76 (95% CI: 1.79–7.92) respectively, with or without detectable anti-HBc. The risk was highest when all three HBV markers (HBsAg, HBV DNA and anti-HBc) were present (OR 46.71, 95% CI: 21.00–103.90).

**Table 2:** Hepatitis B viral markers and the associated odds ratio (OR) for HCC in black South Africans, 2000–2012.

No.	Anti-HBc	HBV DNA	HBsAg	HCC (N = 150) (%)	Controls (N = 437) (%)	OR* (95% CI)
1	-	-	-	27 (18.0)	207 (47.4)	1.00
2	+	-	-	43 (28.7)	175 (40.1)	1.59 (0.90–2.81)
3	-	+	-	6 (4.0)	18 (4.1)	2.60 (0.90–7.53)
4	±	+	-	18 (12.0)	34 (7.8)	3.76 (1.79–7.92)
5	+	+	-	12 (8.0)	16 (3.7)	5.10 (2.06–12.62)
6	-	+	+	7 (4.7)	7 (1.6)	10.19 (2.99–34.75)
7	±	+	+	62 (41.3)	21 (4.8)	34.48 (16.26–73.13)
8	+	+	+	55 (36.7)	14 (3.2)	46.71 (21.00–103.90)

* Adjusted for sex, age group, country and province of birth, number of years lived in urban area, place of birth, alcohol consumption, HIV status and anti-HCV positivity. Occult HBV infection is shown in rows 3–5. Hepatitis B viral marker status -: Negative; +: Positive; ±: Positive and/or Negative.

To investigate the relationship between HBV-DNA levels and HCC development participants were divided into HBV-DNA categories according to the South African guidelines for the management of chronic HBV infection [20] and as shown in Table 3. Of the 150 HCC cases, 80 (53.3%) had detectable levels of serum HBV-DNA, of which 68 (85%) had HBV-DNA levels ≥20,000 and less than 200,000 IU/mL and 41 (51.3%) had greater than or equal to 200,000 IU/mL. Comparable levels in the control participants accounted for 12.8%, 62.5% and 37.5%. An increasing trend in OR was noted with increasing viremia, and was statistically significant from ≥2000 IU/mL (OR 8.55, 95% CI: 3.00–24.54) to ≥200,000 IU/mL (OR 16.93, 95% CI: 8.65–33.13) (Table 3).

**Table 3:** Hepatitis B virus DNA levels and the associated odds ratio (OR) for HCC in black South Africans: 2000–2012.

HBV DNA Levels (IU/ml)	HCC (%)	Control (%)	OR (95% CI)	OR* (95% CI)
≥200,000	41 (27.3)	21 (4.8)	10.65 (5.94–19.11)	16.93 (8.65–33.13)
20,000–199,999	27 (18.0)	14 (3.2)	10.52 (5.26–21.07)	10.97 (5.20–23.14)
2000–19,999	10 (6.7)	8 (1.8)	6.82 (2.60–17.89)	8.55 (3.00–24.54)
200–1999	1 (0.7)	12 (2.7)	0.45 (0.06–3.55)	0.68 (0.08–5.68)
<200	1 (0.7)	1 (0.2)	5.46 (0.34–88.27)	1.54 (0.08–28.22)
Undetected	70 (46.7)	382 (87.2)	1.00	1.00

* Adjusted for sex, age group, country and province of birth, number of years lived in urban area, place of birth, alcohol consumption, HIV status and anti-HCV positivity. P value <0.001.

Table 4 shows the independent and combined effect of HBV and/or HCV, with and without the presence of HIV infection. The excess estimated OR, as risk for HCC development, in individuals with HBV-DNA positivity and HIV infection did not exceed the sum of the relative excess risks for each risk factor alone: 5.36 < (9.09–1.00) + (0.39–1.00). Using our data HBV/HIV co-infection has little effect on risk for the development of HCC. As few HCC and control participants were anti-HCV-positive, the joint effect of HCV with HBV and/or HIV could not be accurately estimated. Irrespective of HBV serological markers HCV-positive participants from the current study were, on average, close to 20 years older than those who were anti-HCV negative (61.9 vs 44.9 years) whereas HBsAg-positive participants were approximately 10 years younger than those who were HBsAg-negative (39.4 vs 46.7 years). HIV-positive HCC cases were significantly older than the HIV-positive controls (43.3 vs. 38.1 years, respectively, p = 0.01). Of the 39 HIV-positive individuals with detectable HBV-DNA, 25 (64.1%) were HBsAg-positive and 14 (35.9%) were HBsAg-negative (p = 0.69). HBV-DNA levels were significantly higher in HBV-HIV co-infected participants, compared to HBV mono-infected—median [IQR] log10 titre: 6.4 (5.5–7.3) vs 5.3 (4.9–6.7), respectively (p = 0.007).

**Table 4:** Main effect of HBV-DNA positivity and interactive effects with anti-HCV- and/or HIV-positivity on the risk for HCC in black South Africans, 2000–2012.

Characteristics	All Participants	HCC	Control	p-value	OR* (95% CI)
	N (%)	N (%)	N (%)		
**HBV DNA and anti-HCV**				<0.001	
HBV DNA-/HCV-	421 (72.3)	52 (34.7)	369 (84.2)		1.00
HBV DNA+/HCV-	129 (22.2)	79 (52.7)	50 (11.4)		11.56 (7.10–18.85)
HBV DNA-/HCV+	28 (4.8)	18 (12.0)	10 (2.3)		10.60 (4.33–26.00)
HBV DNA+/HCV+	4 (0.7)	1 (0.7)	3 (0.7)		3.15 (0.31–32.40)
*Missing*	6 (1.0)	0 (0.0)	6 (1.4)		
**HBV DNA and HIV**				<0.001	
HBV DNA-/HIV-	350 (59.5)	62 (41.3)	288 (65.8)		1.00
HBV DNA+/HIV-	97 (16.5)	60 (40.0)	37 (8.5)		9.09 (5.27–15.66)
HBV DNA-/HIV+	102 (17.3)	8 (5.3)	94 (21.5)		0.39 (0.17–0.89)
HBV DNA+/HIV+	39 (6.6)	20 (13.3)	19 (4.3)		5.36 (2.59–11.11)
*Missing*	0 (0.0)	0 (0.0)	0 (0.0)		
**Anti-HCV and HIV**				<0.001	
Anti-HCV-/HIV-	412 (70.1)	105 (70.0)	307 (70.1)		1.00
Anti-HCV+/HIV-	29 (4.9)	17 (11.3)	12 (2.7)		6.47 (2.69–15.59)
Anti-HCV-/HIV+	138 (23.5)	26 (17.3)	112 (25.6)		0.48 (0.27–0.85)
Anti-HCV+/HIV+	3 (0.5)	2 (1.3)	1 (0.2)		3.69 (0.28–48.36)
*Missing*	6 (1.0)	0 (0.0)	6 (1.4)		
**HBV DNA, anti-HCV and HIV**				<0.001	
No Infection	321 (55.2)	46 (30.7)	275 (63.6)		1.00
HBV DNA only	91 (15.6)	59 (39.3)	32 (7.4)		10.29 (5.90–17.97)
Anti-HCV only	26 (4.5)	16 (10.7)	10 (2.3)		8.98 (3.59–22.46)
HIV only	100 (17.2)	6 (4.0)	94 (21.8)		0.34 (0.14–0.85)
2 or more infection	44 (7.6)	23 (15.3)	21 (4.9)		5.99 (2.99–11.97)
*Missing*	6 (1.0)	0 (0.0)	6 (1.4)		

*Adjusted for sex, age group, country and province of birth, number of years lived in urban area, place of birth, alcohol consumption, HBV DNA, HIV status and anti-HCV positivity

No tested modifiable risk factors showed statistically significant differences between HCC and control groups; approximately half of the HCC participants were non-alcohol drinkers (51.3%) and 47.3% were non-tobacco smokers (Table 5).

**Table 5:** Modifiable risk factor characteristics of HCC cases and of control participants and the associated odds ratio (OR) in black South Africans, 2000–2012.

Characteristics	HCC	Control	p-value	OR (95% CI)	OR* (95% CI)
	N (%)	N (%)			
**Alcohol Use**			0.73		
Non-drinkers	77 (51.3)	225 (51.4)		1.00	1.00
Moderate	46 (30.7)	145 (33.1)		0.93 (0.61–1.41)	1.24 (0.73–1.10)
Heavy	27(18.0)	68 (15.5)		1.16 (0.69–1.94)	1.22 (0.64–2.30)
*Missing Data*	0 (0.0)	0 (0.0)			
**Tobacco Smoking**			0.30		
Non-smokers	71 (47.3)	215 (49.1)		1.00	1.00
Former-smokers	46 (30.7)	121 (27.6)		1.25 (0.74–2.12)	1.63 (0.81–3.29)
Current (1-14g/day)	7 (4.7)	39 (8.9)		1.15 (0.75–1.77)	1.63 (0.85–4.21)
Current (≥15 g/day)	26 (17.3)	63 (14.4)		0.54 (0.23–1.27)	0.62 (0.21–1.89)
*Missing Data*	0 (0.0)	0 (0.0)			
**Diabetes(self-reported)**			0.51		
Negative	145 (96.7)	415 (94.7)		1.00	1.00
Positive	5 (3.3)	23 (5.3)		0.62 (0.23–1.67)	0.83 (0.28–2.50)
*Missing Data*	0 (0.0)	0 (0.0)			
**No. of Lifetime Sexual Partners**			0.57		
0–1	17 (11.3)	48 (11.0)		1.00	1.00
2–5	80 (53.3)	216 (49.3)		1.05 (0.57–1.92)	0.97 (0.46–2.01)
6+	49 (32.7)	165 (37.7)		0.84 (0.44–1.59)	0.81 (0.36–1.82)
*Missing Data*	4 (2.7)	9 (2.1)			
**Hormonal Contraception Use (women only)**			0.09		
Never oral or injectable contraceptive user	13 (8.7)	41 (9.4)		1.00	1.00
Ever Oral and/or injectable contraceptive user	25 (16.0)	73 (16.7)		1.08 (0.50–2.34)	1.09 (0.43–2.79)
*Missing Data*	1 (0.7)	0 (0.0)			

* Adjusted for sex, age group, country and province of birth, number of years lived in urban area, place of birth, alcohol consumption, HBV DNA, HIV status and anti-HCV positivity

## Discussion

Almost a decade has elapsed since the most recent investigation of risk factors for and demographics of HCC in SA [22]. Consistent with previous hospital-based case-controls studies [22–26], we found a mean age of 46 years and a predominance of men in the HCC cases. Although our results show a higher prevalence of previous exposure to HBV in rural-born than in urban-born HCC cases (65% vs 35%, p<0.001), when compared to an earlier study in the 1980s [26], the lack of statistical significance in HBsAg positivity between rural- and urban-born cases was consistent (p = 0.3). Thus, HCC participants born in urban environments are just as likely to have been infected with HBV during early childhood as those who were born and in rural areas and subsequently moved to an urban area [27]. Although not investigated in the present study, it is possible that those born in rural areas are also vulnerable to co-carcinogens prevalent in rural settings, such as dietary exposure to aflatoxin B_1_ and iron overload [28, 29]. When they leave the rural areas for urban environments, their exposure to these co-carcinogens may decrease, thereby delaying the progression of HCC [27].

HBsAg prevalence in our study as a whole (14%) and in the HCC participants (41%), are in agreement with the prevalence found in the general SA population (from 8% to 10%) [30] and in HCC patients from previous SA case-controls studies (ranging from 40.3% to 70%) [27, 30–32]. Our study shows that in black South Africans HBV infection continues to be a primary risk factor for HCC development, followed by HCV infection. Our risk estimates for HBsAg (OR 34.5) and anti-HCV (OR 9.0) are consistent with a study of HCC patients attending a rural hospital in Mpumalanga, SA (OR 33.2 for HBsAg) [31], but are somewhat higher than another study conducted in urban Johannesburg hospitals (OR 23.3 for HBsAg and 6.6 for anti-HCV) [32]. The synergistic effect of HBV-HCV co-infection for risk of developing HCC observed by Kew *et al*. [32], was not found in the current analysis (Table 4), possibly because very few participants were infected with both viruses (Table 4).

To the best of our knowledge the current study is the first to determine an association between HCC risk and the 2000 IU/mL threshold of HBV-DNA levels in black South Africans, as well as the increased risk of HCC with increasing HBV-DNA level, in a dose-response relationship (Table 3). We found only one prior study that estimated HCC risk related to HBV-DNA viral load in SA: among 124 HBsAg-positive cases and 125 asymptomatic carriers identified in a cohort of black southern Africans, the reported geometric mean viral loads were 553,618 copies/ml (approximately 118,000 IU/mL) and 16,084 copies/ml (approximately 3,400 IU/mL), respectively [22]. In the current study the risk of developing HCC in individuals with low HBV-DNA levels (<2000 IU/mL) was not significant, and differed in comparison to earlier studies conducted in Korea [33] and The Gambia [34] where five- and three-times higher respective risks were reported. This discrepancy, observed at low HBV-DNA levels, may result from marked differences in population characteristics and HBV genotypes both of which may influence the viral load. Moreover, environmental risk factors in West Africa such as aflatoxin B_1_[35]—which is classified as a human carcinogen by IARC (International Agency for Research on Cancer)—may play an important role, especially at low viral loads. In the 1980s aflatoxin contamination in SA commercial foodstuffs was shown to be low (0.66 μg/kg) in comparison to Mozambique (range from 0.7–6.43 μg/kg) [36]; this was also confirmed in a more recent study (2004) that reported low rates of mycotoxin contamination in staple foods such as maize [37]. In any case suppression of HBV, even if only for a finite period, may significantly reduce risk for developing HCC [38]. Long-term follow-up studies are needed to further determine the role of low HBV-DNA levels as a risk factor for HCC in black South Africans.

In the current study, although the HIV prevalence in the case (18.7%) and control (25.8%) groups did not differ significantly (p = 0.08), both groups were higher than the background HIV seropositivity in this adult population in Gauteng province, SA (12.4%) [39]. Furthermore, we did not find an increased risk of developing HCC in relation to HIV infection (OR 0.4). This is consistent with an earlier case-control study from SA in the 1990s, that recruited 913 black African HIV cases and 325 controls comprised of patients with cancers other than those known to have an infectious cause (OR 0.9, 95% CI:0.3–2.9) [14]. Although it has been suggested that HIV infection accelerates liver damage related to HBV infection [40], a possible explanation for our finding a lack of higher HCC risk in HIV-positive individuals might be the reduction in the continual inflammation related to immune-mediated clearance of HBV-infected hepatocytes [41] as a result of impaired innate and adaptive immune response in HIV-coinfection [42]. Additionally, given the long induction period of HCC, many South African HIV-infected patients, in the absence of ART, died earlier, before symptoms related to HBV could manifest and in contrast to evidence from developed countries [43, 44]. Millions of relatively young HIV-infected SA adults are now surviving longer as a result of the widespread rollout of ART which began in 2009 [45] and is now available free of charge to all HIV infected people, regardless of CD4 count [46]. This may or may not be associated with a parallel increase in the burden of HCC depending on the coverage of infant HBV vaccination, the possible spread of HCV and also an increased prevalence of modifiable risk factors [47]. The shift in cancer burden from AIDS-defining cancers to non-AIDS defining cancers seen in developed countries is not yet evident in SA but is likely to occur in time [48]. Although both HIV and HBV are endemic in SA systematic studies investigating the association between HBV-HIV coinfection and HCC have not been done. This may reflect under-ascertainment and reporting due to the often late presentation with cancer, especially in resource-limiting settings thus further highlighting the need for a systematic approach to surveillance for HCC in the HIV-positive population in SA [4, 48].

OBI prevalence found in the HCC cases (12%) was significantly less than that found by an earlier SA study (48.4%) [25] but translated to a four-times higher risk of HCC (Table 2). Our findings show that OBI is associated with increased HCC risk (Table 2), and the highest OR of 5.10 (95% CI: 2.06–12.62) in OBI-infected participants with anti-HBc positivity was consistent with a previous meta-analysis of retrospective studies (OR 6.08, 95% CI: 3.45–10.72) [49]. Although the clinical implications of OBI are not clearly understood, this condition has been extensively reported in HIV-infected ART-naïve patients in SA, with a prevalence ranging from 15.1% to 23% [16, 50], higher than that found in the current study (8.8%). The differences may be influenced by the ART use. Although OBI did not differ between HIV-positive and negative individuals in this study (9.9% vs 8.5%, p = 0.238), the presence of OBI poses a unique challenge in managing HBV disease, particularly against a backdrop of the high HIV prevalence in the general SA population (12% or 7 million are HIV-positive) (http://aidsinfo.unaids.org). Future research is required to determine the true risk of acquiring OBI as it is not detected by conventional HBsAg testing.

This study has limitations. Modifiable risk factors were self-reported, there was limited data on the prior use of antiretroviral treatment, absence of HIV viral and CD4 count, and an absence of sufficient clinical data evaluating liver function and/or the presence of cirrhosis, all of which may have influenced the estimates of HCC risk. Additionally, no aflatoxin exposure or HCV virological data were available. Using sera following prolonged storage could potentially introduce errors as a result of protein or HBV-DNA degradation. However, this possible effect was minimized by selecting control participants that were matched by time and hospital to the HCC cases enrolled. While the use of sonography in detecting HCC has its limitations [51], only four included cases were diagnosed by this method alone. In limited-resource settings HCC patients often present at an advanced stage where the finding of a mass using imaging techniques is rarely confirmed by histological examination [52].

A considerable proportion of HCC burden in northern SA is being borne by rural migrants to urban areas, the majority of whom are working-age men who may have had exposure to co-carcinogens prevalent in the rural environment. HBV-induced HCC will continue to occur in the older population group and in immigrants, and may increase in the foreseeable future especially when HIV co-infected individuals have an extended lifespan due to ART therapy, thus allowing sufficient time for HCC to develop [53]. Given the overburdened public health services in SA, the challenge is to put in place preventative measures through health education and early detection and screening programs targeting high risk individuals residing in rural areas. Poverty alleviation and the provision of cost-effective medical care will also assist in HCC treatment and in incidence reduction.
